# Integrated Psychological–Behavioral Predictive Model Using Explainable Machine Learning

**DOI:** 10.3390/healthcare14172777

**Published:** 2026-09-01

**Authors:** Ali Mohammed Abuhekmah, Yahya Mubark Khatatbeh

**Affiliations:** 1 Abha Mental Health Hospital, Ministry of Health, Abha 62585, Saudi Arabia; 2 Department of Psychology, College of Social Sciences, Imam Mohammad Ibn Saud Islamic University (IMSIU), Riyadh 11564, Saudi Arabia

**Keywords:** medication adherence, mental health literacy, medication beliefs, machine learning, explainable artificial intelligence, psychiatry

## Abstract

**Background:** Medication non-adherence remains a major challenge in psychiatric care, yet its prediction often relies on clinical and sociodemographic characteristics, while giving comparatively limited attention to modifiable psychological–cognitive factors. Integrating mental health literacy and medication-related beliefs with explainable machine learning approaches may provide a more informative framework for understanding and predicting adherence. **Objective:** This study examined the association of mental health literacy and medication-related beliefs with psychotropic medication adherence and evaluated their explanatory and predictive values using conventional statistical modeling, machine learning, explainable artificial intelligence, and structural path analysis. **Methods:** A cross-sectional study was conducted with 225 psychiatric patients. Medication adherence; mental health literacy; and beliefs about medication necessity, concerns, harm, and overuse were assessed using self-report measures, including a MARS-derived eight-item adherence measure. Associations were examined using Spearman correlations, hierarchical regression with robust inference, and a secondary observed-variable path representation of multivariable associations with 5000 bootstrap resamples. The Elastic Net, Random Forest, and Gradient Boosting models were evaluated using repeated five-fold cross-validation. Shapley Additive exPlanations (SHAP) were used to interpret the best-performing model. **Results:** Greater adherence was associated with higher mental health literacy (ρ = 0.361) and stronger necessity beliefs (ρ = 0.353), whereas concern (ρ = −0.474), perceived harm (ρ = −0.528), and overuse beliefs (ρ = −0.284) were negatively associated with adherence (all *p* < 0.001). The psychological–cognitive model explained 40.0% of the variance in the primary eight-item adherence composite. The integrated Random Forest achieved the best out-of-sample performance (R^2^ = 0.402, MAE = 0.958, RMSE = 1.299; *n* = 208), although the improvement over the psychological–cognitive Random Forest (R^2^ = 0.375) was modest. SHAP identified perceived harm and medication concerns as leading predictors. **Conclusions:** Medication adherence in psychiatric patients was more strongly characterized by psycho-cognitive factors than by sociodemographic and clinical characteristics alone. Mental health literacy, necessity beliefs, medication concerns, and perceived harm may represent particularly informative targets for individualized adherence assessments and future intervention development. Prospective external validation is required before clinical implementation.

## 1. Introduction

Adherence to medication remains a major challenge for the long-term management of psychiatric disorders. Pharmacotherapy is central to the treatment of schizophrenia spectrum disorders, bipolar disorder, major depressive disorder, and other persistent psychiatric conditions. However, its effectiveness depends substantially on patient adherence to the prescribed regimens. Medication non-adherence remains highly prevalent, with a systematic review and meta-analysis estimating an overall rate of approximately 49% among patients with major psychiatric disorders, including 56% in schizophrenia, 50% in major depressive disorder, and 44% in bipolar disorder [1,2,3,4]. Poor adherence is associated with inadequate symptom control, delayed recovery, increased relapse risk, and greater healthcare utilization, making it a persistent clinical challenge in psychiatric care [1,2,5]. Medication adherence is increasingly conceptualized as a multifaceted health behavior rather than simply as treatment compliance. Clinical and treatment-related factors, adverse effects, therapeutic relationships, social support, illness perceptions, and psychological factors may influence medication-taking behavior [1,3,6,7]. Importantly, patients actively appraise the expected benefits and potential harm of pharmacotherapy and develop beliefs about its necessity, safety, and appropriate use. Consequently, patients with comparable diagnoses and treatment regimens may exhibit substantially different adherence patterns, highlighting the importance of potentially modifiable psychological and cognitive determinants [5,8,9].

One potentially modifiable determinant of medication adherence is mental health literacy (MHL), a multidimensional construct encompassing the knowledge and beliefs required to recognize, manage, and prevent mental disorders; understand available treatments; access professional support; and engage in appropriate help-seeking. In Saudi Arabia, BinDhim and Althumiri [10] validated an Arabic Mental Health Literacy Scale in 544 adults, reporting good internal consistency (α = 0.87) and test–retest reliability (ICC = 0.89). Recent evidence has also emphasized medication-related literacy and patients’ capacity to obtain, understand, and appropriately use medication information [9,11,12]. However, knowledge alone may not translate directly into medication adherence because treatment decisions are also shaped by patients’ cognitive appraisals of pharmacotherapy. Medication-related beliefs may, therefore, constitute an important mechanism linking treatment understanding with medication-taking behavior [13,14].

Mental health literacy, medication-related literacy, and medication beliefs are conceptually distinct constructs. Mental health literacy broadly refers to knowledge and beliefs that facilitate the recognition, management, and prevention of mental disorders, whereas medication-related literacy concerns an individual’s ability to obtain, understand, and appropriately use information specifically related to medicines [15]. In contrast, medication beliefs reflect patients’ cognitive appraisals of treatment, including perceived necessity, concerns, harm, and overuse. These constructs were considered within the same framework because understanding mental health and treatment information may shape, but is not equivalent to, patients’ evaluations of prescribed medication; together, they may provide complementary perspectives on medication-taking behavior [14].

The Beliefs about Medicines Questionnaire (BMQ) distinguishes treatment-specific beliefs about medication necessity and concerns from general beliefs regarding medication harm and overuse [14,16,17]. Although conceptually related, these dimensions represent distinct appraisals and are therefore more appropriately examined separately than a single global attitude toward medication [16,18,19]. The relationship between treatment-specific beliefs and adherence is theoretically grounded in the Necessity–Concerns Framework (NCF), which proposes that medication-taking decisions reflect patients’ appraisals of their personal need for treatment relative to their concerns about its potential adverse consequences. In a meta-analysis of 94 studies involving 25,072 participants, stronger necessity beliefs were associated with greater adherence (OR = 1.742, 95% CI [1.569, 1.934]), whereas greater concerns were associated with poorer adherence (OR = 0.504, 95% CI [0.450, 0.564]) [14]. A subsequent meta-analysis similarly reported significant associations for necessity (r = 0.17), concerns (r = −0.18), and necessity–concerns differential (r = 0.24), indicating that medication beliefs meaningfully contribute to adherence while operating alongside other determinants [16,20].

NCF is particularly relevant to psychotropic treatment, in which patients’ perceived need for long-term medication may coexist with concerns regarding adverse effects, dependence, daily functioning, and potential long-term harm. Evidence from psychiatric populations indicates that stronger necessity beliefs are generally associated with better medication adherence, whereas greater concerns and negative medication beliefs are associated with poorer adherence [6,8,9]. General beliefs regarding medication harm and overuse may provide additional information because patients may simultaneously perceive medicines in general as harmful or overprescribed, while regarding their own psychotropic medication as necessary. Examining necessity, concerns, harm, and overuse as distinct dimensions may therefore provide a more differentiated account of medication-taking behavior [8,14,18].

These issues are particularly relevant to psychiatric care in Saudi Arabia. In a recent study of 260 patients with mood disorders attending a Saudi tertiary care hospital, 79.2% demonstrated good medication adherence, which was associated with treatment satisfaction, patient–doctor relationship, illness duration, age, and the number of regularly prescribed medications [21,22]. Saudi research has also demonstrated the feasibility of assessing MHL within the local cultural and linguistic context and has documented increasing attention to medication literacy [10,11]. However, these lines of research have largely been developed independently, and limited evidence has examined mental health literacy and multidimensional medication beliefs simultaneously in relation to medication adherence among Saudi psychiatric patients.

Beyond identifying factors associated with medication adherence, an important clinical question is whether adherence can be predicted at the individual level by integrating psychological, cognitive, sociodemographic, and clinical information. Conventional explanatory analyses clarify the direction and magnitude of associations, whereas predictive modeling can evaluate how well these factors jointly distinguish individual differences in adherence. In psychiatric care, combining these perspectives may therefore provide complementary insights into both the factors associated with adherence and their potential predictive relevance [6,10,14]. However, relatively few studies have integrated mental health literacy and multidimensional medication beliefs within a unified explanatory and predictive framework, particularly among psychiatric patients in Saudi Arabia. This gap limits the understanding of whether these theoretically relevant psychological–cognitive factors provide complementary information for explaining and predicting medication adherence.

Accordingly, the present study examined medication adherence within an integrated psychological–cognitive framework encompassing mental health literacy and four medication belief dimensions—necessity, concerns, perceived harm, and perceived overuse—alongside relevant sociodemographic and clinical characteristics. This study combined explanatory and predictive perspectives to evaluate the associations of these factors with medication adherence, their relative contribution to prediction, and their simultaneous relationships within an integrated conceptual framework. This approach was intended to provide a more comprehensive understanding of medication adherence while maintaining a clear distinction between explanatory association and predictive performance.

RQ1. What are the levels and distributions of medication adherence, mental health literacy, and medication-related beliefs among psychiatric patients?

RQ2. What are the associations between mental health literacy and medication-related beliefs and medication adherence, and to what extent do these psychological and cognitive factors explain the variability in medication adherence?

RQ3. To what extent can medication adherence be predicted using psychological–cognitive, sociodemographic, and clinical characteristics, and does an integrated machine-learning model improve out-of-sample predictive performance?

RQ4. Which psychological, cognitive, sociodemographic, and clinical factors contribute most strongly to machine learning predictions of medication adherence, and how do they influence model predictions?

RQ5. Are the associations between mental health literacy and medication adherence statistically accounted for, in part, by medication-related beliefs?

## 2. Materials and Methods

### 2.1. Study Design and Setting

This observational study employed an analytical cross-sectional design to examine the associations of mental health literacy and medication-related beliefs with medication adherence among psychiatric patients and to evaluate the complementary predictive contribution of psychological–cognitive, sociodemographic, and clinical characteristics. The analytical framework incorporated descriptive and correlational analyses, hierarchical multiple regression, supervised machine learning prediction with explainable model interpretation, and observed-variable structural path analysis. These approaches have been used to provide complementary associational, explanatory, predictive, and structural perspectives regarding medication adherence.

The study was conducted at the Abha Psychiatric Hospital in the Aseer Region of Saudi Arabia, a specialized mental health setting that provides psychiatric assessment, follow-up care, and pharmacological treatment. Participants were recruited from psychiatric patients receiving ongoing clinical care and prescribed psychotropic medication in the study setting.

### 2.2. Participants and Eligibility Criteria

Participants were adult psychiatric patients receiving outpatient or follow-up care at the Abha Psychiatric Hospital. A total of 225 participants met the eligibility criteria, provided informed consent, and were included in this study.

Participants were eligible if they (1) were aged 18 years or older, (2) had a documented psychiatric diagnosis established by the treating clinical team, (3) were currently prescribed at least one psychotropic medication, (4) were receiving ongoing pharmacological treatment at the time of data collection, (5) were clinically stable at the time of assessment and able to participate in the study procedures, and (6) were able to understand the study information and provide informed consent.

Participants were excluded if they (1) experienced an acute psychiatric episode or clinical instability requiring immediate intensive intervention; (2) had cognitive, neurological, intellectual, or communication difficulties of sufficient severity to preclude reliable completion of the study measures; (3) were unable to provide informed consent; or (4) returned questionnaires with insufficient data for the primary study variables.

Primary statistical analyses were conducted using a full analytical sample of 225 participants. For analyses requiring an expanded set of sociodemographic and clinical predictors, the analytic sample was restricted to participants with complete data for all variables included in the relevant model. The sample size used for each analysis was reported along with the corresponding analysis.

### 2.3. Study Measures

#### 2.3.1. Sociodemographic and Clinical Information Form

A structured participant information form was used to collect sociodemographic and clinical characteristics. Sociodemographic variables included age, sex, marital status, educational level, employment status, perceived economic level, and place of residence. Clinical and treatment-related variables included psychiatric diagnosis, duration of psychiatric disorders, number of prescribed psychotropic medications, perceived family support, and regularity of treatment follow-up. These variables were used to characterize the study sample and, where applicable, as contextual predictors in the machine learning analyses.

The sociodemographic and clinical information form was developed specifically for the present study to capture participant characteristics relevant to medication adherence analyses. The selected variables were defined a priori based on the study objectives and commonly examined sociodemographic and clinical correlates of medication-taking behavior. The form was used solely to characterize the sample and to provide contextual predictors for the statistical and machine-learning analyses; it was not intended to constitute a psychometric scale or generate a composite score.

#### 2.3.2. Medication Adherence Measure

Medication adherence was assessed using an eight-item measure derived from the 10-item Medication Adherence Rating Scale (MARS), originally developed by Thompson et al. [23] to assess medication-taking behavior and attitudes toward psychotropic medication. The original MARS is a 10-item dichotomous (Yes/No) self-report instrument derived from the Drug Attitude Inventory and Medication Adherence Questionnaire, with total scores ranging from 0 to 10, with higher scores indicating better medication adherence. The original instrument encompasses three conceptual components: medication adherence behavior, attitudes toward taking medication, and negative attitudes and experiences related to psychotropic medication.

The instrument administered in the present study comprised eight of the ten original MARS items. Specifically, original MARS Item 3 (“When you feel better do you sometimes stop taking your medication?”) and Item 4 (“Sometimes if you feel worse when you take the medication do you stop taking it?”) were not included in the administered study instrument and, consequently, were not available in the dataset. These omissions occurred at the instrument-administration stage and were not the result of post-hoc item deletion based on the present psychometric analyses. Because the contemporaneous study documentation did not provide a specific item-level rationale for their omission, no retrospective theoretical or psychometric justification was attributed to their exclusion. To ensure transparency and avoid treating the administered instrument as equivalent to the validated 10-item MARS, it is therefore referred to throughout this study as the “MARS-derived eight-item adherence measure”.

The eight administered items were coded in an adherence-consistent direction and summed to produce a continuous total score ranging from 0 to 8, with higher scores indicating greater adherence-related responses. The composite contained both medication-taking behavioral content and attitudinal/experiential content related to medication. Accordingly, the continuous eight-item score was retained as the primary adherence-related outcome, and no categorical adherence cutoff derived from the original 10-item MARS was applied. The internal consistency of the eight-item measure in the present sample was relatively low (KR-20/Cronbach’s α = 0.490). Item-level psychometric analyses were therefore conducted, including corrected item–total correlations and α-if-item-deleted coefficients.

Given that the original MARS Items 3 and 4 form part of the behavioral adherence component of the original instrument, additional sensitivity analyses were undertaken to distinguish medication-taking behavior from the broader attitudinal and experiential content of the eight-item composite. Therefore, a separate behavioral adherence score was calculated using the two available behavioral items assessing medication forgetting and carelessness. This two-item score was used only as a sensitivity outcome and was not treated as a replacement for the primary eight-item composite. These analyses were intended to evaluate the robustness of the principal findings to the outcome definition and to examine the potential influence of construct overlap between the primary adherence-related composite and medication belief predictors.

#### 2.3.3. Mental Health Literacy Measure

Mental health literacy was assessed using the 16-item Mental Health Literacy Questionnaire–Short Version for Adults (MHLq-SVa) developed and cross-culturally validated by Campos et al. [24]. The instrument comprises four dimensions: knowledge of mental health problems (six items), erroneous beliefs/stereotypes (three items), help-seeking and first-aid skills (three items), and self-help strategies (four items). Responses were recorded on a five-point Likert scale ranging from 1 (strongly disagree) to 5 (strongly agree). The three negatively worded erroneous-belief items were reverse-scored, such that higher values consistently represented greater mental health literacy. The item scores were summed to produce a total score ranging from 16 to 80, with higher scores indicating greater mental health literacy. In the present sample, the total MHLq-SVa demonstrated acceptable internal consistency (Cronbach’s α = 0.797). The internal consistency coefficients for the four dimensions were 0.756 for knowledge of mental health problems, 0.626 for erroneous beliefs/stereotypes, 0.692 for help-seeking and first-aid skills, and 0.674 for self-help strategies. The complete item wording and scoring directions are provided in Supplementary Table S1.

#### 2.3.4. Beliefs About Medicines Questionnaire (BMQ)

Medication-related beliefs were assessed using the Beliefs About Medicines Questionnaire (BMQ), developed by Horne and Weinman [17]. The BMQ is designed to assess patients’ cognitive representations of medication and comprises treatment-specific and general belief domains. The present study examined four dimensions: necessity beliefs, reflecting perceived personal need for prescribed medication; medication concerns, reflecting concerns about the potential adverse consequences of treatment; perceived medication harm, reflecting general beliefs that medicines may be harmful; and perceived medication overuse, reflecting beliefs that medicines are excessively prescribed or relied upon. Items were scored according to the standard response format, with higher scores indicating stronger endorsement of the corresponding belief dimension. Each dimension was analyzed separately rather than combined into a global medication belief score, consistent with the multidimensional conceptualization of the BMQ and the Necessity–Concerns Framework [14,17]. In the present sample, internal consistency was good across the four BMQ dimensions: necessity beliefs (Cronbach’s α = 0.896), medication concerns (α = 0.779), perceived medication harm (α = 0.814), and perceived medication overuse (α = 0.728).

### 2.4. Data Collection Procedure

Data were collected from February to May 2026 at the Abha Psychiatric Hospital in the Aseer Region. Data were collected from February to May 2026 at Abha Psychiatric Hospital in the Aseer Region of Saudi Arabia, following ethical approval from the Aseer Institutional Review Board. Potential participants were consecutively approached and screened according to the prespecified inclusion and exclusion criteria. A total of 253 patients were assessed for eligibility; 28 were excluded because they did not meet the eligibility criteria, resulting in a final analytical sample of 225 participants. Eligible participants received information about the study objectives and procedures, the voluntary nature of participation, confidentiality, and their right to withdraw at any time without affecting the healthcare services they received. Written informed consent was obtained prior to participation in the study.

**Figure 1:**
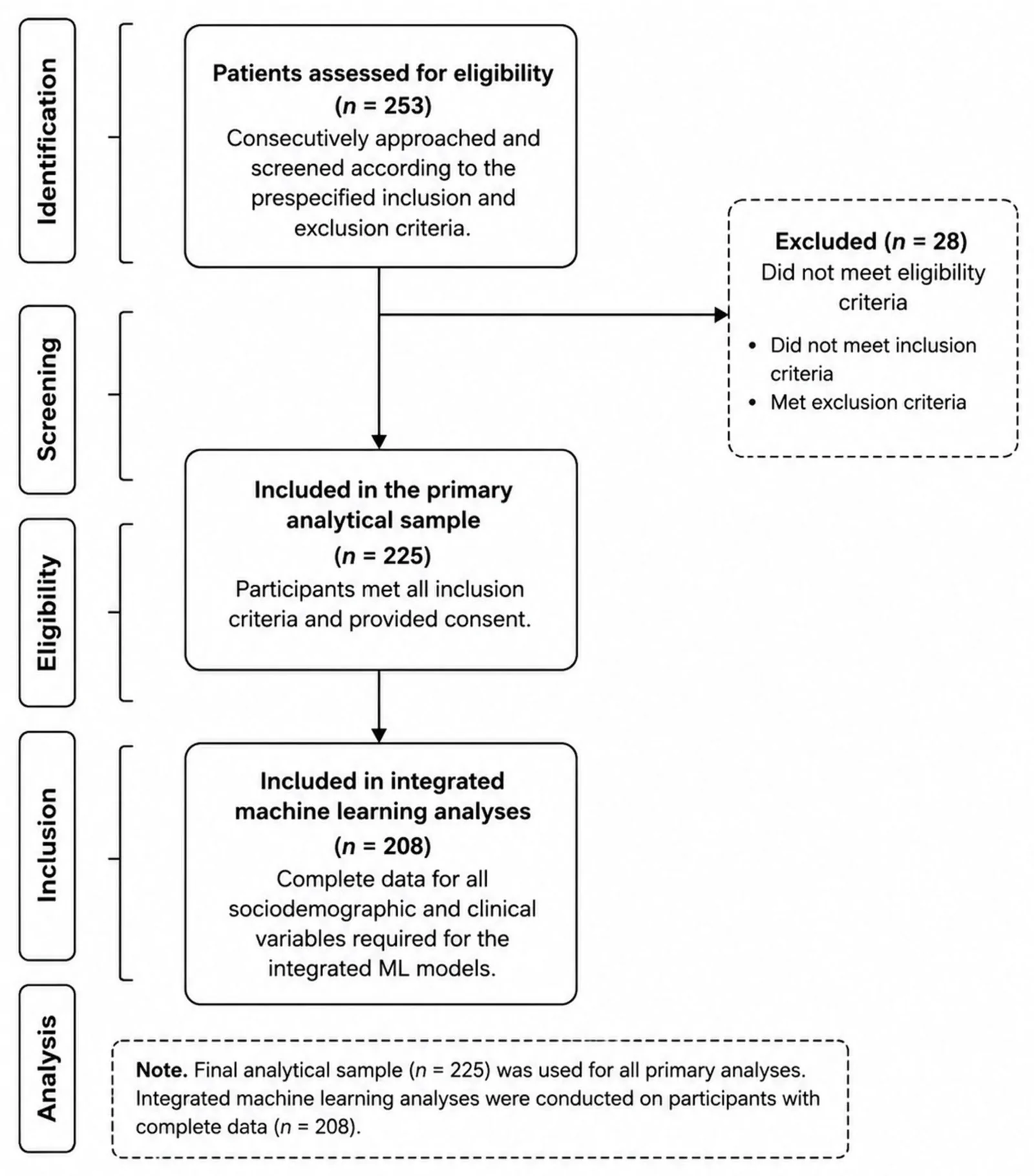
Participant flow through eligibility assessment and analytical samples.

The questionnaires were administered under the supervision of the first author in coordination with the hospital and relevant clinical staff. Treating clinicians were not present while participants completed the questionnaires to reduce potential social-desirability and response-pressure effects. Participants completed the measures individually, with assistance limited to the clarification of standardized instructions when required. Data collection was performed in the clinical setting at times that did not interfere with routine patient care. Participants completed the sociodemographic and clinical information form, medication adherence measure, mental health literacy measure, and Beliefs About Medicines Questionnaire (BMQ). Standardized instructions were provided, and completion required approximately 15–20 min. The completed questionnaires were reviewed for completeness before coding and data entry. Subsequently, the dataset was checked for completeness, coding accuracy, and implausibility. Participant identifiers were removed from the analytical dataset, and confidentiality was maintained throughout data management, analysis, and reporting. The participant flow through eligibility screening and inclusion in the primary and machine-learning analytical samples is shown in Figure 1.

### 2.5. Ethical Considerations

Ethical approval was obtained from the Aseer Institutional Review Board (Aseer IRB), Ministry of Health, Kingdom of Saudi Arabia (Approval No. IRB-169-2025; National Registration No. H-06-B-091) on 11 February 2026, before the commencement of data collection. This study was conducted in accordance with the approved protocol and the applicable institutional and national ethical requirements for research involving human participants. Written informed consent was obtained from all participants before data collection. Participation was voluntary, and participants were informed of their right to withdraw without consequences for their healthcare. Confidentiality and privacy were maintained throughout the study, and no personally identifiable information was included in the analytical dataset.

### 2.6. Statistical Analysis

Descriptive statistics were used to summarize the participant characteristics and study variables. Internal consistency was assessed using Cronbach’s α or KR-20, as appropriate. Statistical methods were selected to address complementary analytical objectives. Spearman correlations assessed bivariate associations, hierarchical regression evaluated incremental explanatory contributions, machine learning models assessed out-of-sample prediction, and structural path analysis examined simultaneous relationships among the principal study variables. Associations between medication adherence and psychological–cognitive variables were examined using Spearman’s correlations with 95% confidence intervals estimated from 5000 bootstrap resamples. Hierarchical multiple regression analysis was used to estimate the incremental contribution of medication-related beliefs beyond mental health literacy, with R^2^, adjusted R^2^, and ΔR^2^ reported. HC3 heteroskedasticity-consistent standard errors were used for coefficient inference, and multicollinearity and residual diagnostics were examined.

Given the low internal consistency of the MARS-derived eight-item adherence measure, corrected item–total correlations and KR-20/Cronbach’s α if item deleted were calculated. To examine potential construct overlap with medication-belief predictors, correlation and HC3 regression analyses were repeated using a two-item behavioral adherence sensitivity score based on medication forgetting and carelessness, with higher scores indicating better behavioral adherence.

For machine-learning analyses, Elastic Net, Random Forest, and Gradient Boosting regression models were evaluated using psychological–cognitive, sociodemographic/clinical, and integrated predictor sets. For a direct comparison across feature sets, analyses were conducted on 208 participants with complete sociodemographic and clinical information. Continuous predictors were median-imputed when required and standardized within the training data, whereas categorical predictors were imputed using the most frequent category and one-hot encoding. All pre-processing operations were performed exclusively within the training folds to prevent information leakage.

Model development and internal validation used repeated five-fold cross-validation with five repeats. Hyperparameter tuning was conducted exclusively within the training data of the cross-validation procedure, and all preprocessing and model selection steps were restricted to the training folds to minimize information leakage. A separate nested cross-validation framework was not implemented; accordingly, the reported performance estimates should be interpreted as internal cross-validated predictive performance rather than external or prospective validation. Elastic Net was tuned over α = (0.01, 0.10, 1.00) and L1 ratios = (0.25, 0.50, 0.75); Random Forest over 100 or 200 trees, maximum depth = (None, 5, 10), maximum features = (sqrt, 0.50), and minimum leaf size = (1, 3, 5); and Gradient Boosting over 100 or 200 estimators, learning rates = (0.03, 0.10), maximum depth = (1, 2, 3), minimum leaf size = (1, 5), and subsampling fractions = (0.80, 1.00). Candidate configurations were selected according to the cross-validated R^2^.

Predictive performance was evaluated using cross-validated R^2^, mean absolute error (MAE), and root mean squared error (RMSE), with performance estimates averaged across repeated validation folds. The resulting estimates represent internal cross-validated predictive performance rather than prospective or externally validated clinical predictions. The best-performing model was interpreted using SHapley Additive exPlanations (SHAP).

Finally, an observed-variable path analysis was used only as a secondary graphical representation of the simultaneous associations between mental health literacy, medication belief dimensions, and medication adherence. Standardized path coefficients, explained variance (R^2^), and 95% confidence intervals (CIs) based on 5000 bootstrap resamples are reported. Because the model was just identified and reproduced the associations evaluated in the multivariable regression, it was not interpreted as independent confirmatory evidence, and global model-fit indices were not interpreted. The primary inferential conclusions were based on regression and sensitivity analyses.

### 2.7. Reporting Guidelines

This study was conducted in accordance with the Strengthening the Reporting of Observational Studies in Epidemiology (STROBE) statement for its cross-sectional observational component and the TRIPOD+AI statement for the reporting of the machine-learning prediction component.

## 3. Results

The results are presented according to the five research questions. Descriptive analyses were reported first, followed by bivariate associations and multivariable regression analyses. The subsequent sections present the out-of-sample machine learning prediction results, SHAP-based model interpretation, and structural path analysis. This sequence provides a progressive evaluation of the descriptive, explanatory, predictive, and structural dimensions of medication adherence.

### 3.1. Participant Characteristics

Participant Characteristics. Table 1 summarizes the sociodemographic and clinical characteristics of the 225 participants. The largest age group was 31–36 years (28.0%), followed by 37–42 years (26.2%). Males constituted 59.1% of the sample, and most participants were single or married. Secondary education and university education were the predominant educational levels, and approximately half of the participants were unemployed (50.2%). Most participants reported moderate economic status (58.7%) and lived in urban areas (70.2%). Depressive and mood disorders constituted the largest diagnostic group (47.1%), followed by anxiety disorders (23.1%). Most participants reported family support (66.2%) and regular treatment follow-up (87.1%).

RQ1. What are the levels and distributions of medication adherence, mental health literacy, and medication-related beliefs among psychiatric patients?

As shown in Table 2, the mean medication adherence score was 5.25 (SD = 1.70), with scores spanning the full 0–8 range. Mental health literacy had a mean score of 67.76 (SD = 8.12) and showed a negatively skewed distribution (skewness = −1.30), indicating greater concentration toward the upper end of the scale. Medication-belief scores also varied across dimensions, with mean scores of 16.72 (SD = 5.22) for necessity, 16.91 (SD = 4.93) for concerns, 11.99 (SD = 4.11) for perceived harm, and 8.72 (SD = 2.67) for perceived overuse.

**Table 2:** Descriptive Statistics for Medication Adherence, Mental Health Literacy, and Medication-Related Beliefs.

Variable	Possible Range	Mean	SD	Median	IQR	Observed Range	95% CI for Mean
Medication adherence (MARS-derived 8-item measure)	0–8	5.25	1.70	5.00	4.00–6.00	0–8	5.03–5.48
Mental health literacy	16–80	67.76	8.12	69.00	63.00–74.00	28–80	66.70–68.83
Medication necessity beliefs	5–25	16.72	5.22	17.00	14.00–20.00	5–25	16.03–17.40
Medication concerns	6–30	16.91	4.93	17.00	14.00–20.00	6–30	16.27–17.56
Perceived medication harm	5–25	11.99	4.11	12.00	9.00–15.00	5–25	11.45–12.53
Perceived medication overuse	3–15	8.72	2.67	9.00	7.00–10.00	3–15	8.38–9.07

Note. *n* = 225. Higher scores indicate greater medication adherence, mental health literacy, and stronger endorsement of the corresponding medication belief dimension. The MARS-derived measure comprised eight items; therefore, no categorical adherence cutoff was applied.

Item-level psychometric analysis showed corrected item–total correlations ranging from 0.105 to 0.309. The internal consistency of the MARS-derived 8-item measure was low (KR-20/Cronbach’s α = 0.490). Deletion of individual items did not materially improve reliability, with α-if-item-deleted coefficients ranging from 0.418 to 0.504. The highest coefficient obtained after the deletion of any single item was 0.504, indicating that the low internal consistency was not attributable to a single poorly performing item. Given the mixed behavioral and attitudinal/experiential content of the composite, additional sensitivity analyses were conducted using the available two-item behavioral adherence score.

#### Behavioral Adherence Sensitivity Analysis

To examine whether the associations observed for the primary MARS-derived 8-item composite were influenced by its attitudinal/experiential content, sensitivity analyses were conducted using the available two-item behavioral adherence score assessing medication forgetting and carelessness. The behavioral adherence score had a mean of 1.11 (SD = 0.79). The two behavioral items were moderately correlated (r = 0.425), with a Spearman–Brown reliability coefficient of 0.596. Bivariate associations were generally directionally consistent with those obtained using the primary eight-item composite but were substantially attenuated. Behavioral adherence was positively associated with mental health literacy (ρ = 0.132, *p* = 0.048) and necessity beliefs (ρ = 0.131, *p* = 0.049) and negatively associated with medication concerns (ρ = −0.184, *p* = 0.006) and perceived medication overuse (ρ = −0.233, *p* < 0.001). The association with perceived medication harm was negative but did not reach conventional statistical significance (ρ = −0.128, *p* = 0.055).

In the multivariable sensitivity analysis using HC3 heteroskedasticity-consistent standard errors, the psychological–cognitive predictors explained 8.0% of the variance in the behavioral adherence score (R^2^ = 0.080, adjusted R^2^ = 0.059) compared with 40.0% of the variance in the primary eight-item composite (R^2^ = 0.400). Perceived medication overuse remained independently associated with poorer behavioral adherence (β = −0.185, 95% CI [−0.340, −0.030], *p* = 0.019). Mental health literacy (β = 0.104, *p* = 0.136), necessity beliefs (β = 0.116, *p* = 0.118), medication concerns (β = −0.147, *p* = 0.065), and perceived medication harm (β = 0.103, *p* = 0.287) were not independently significant in the behavioral sensitivity model.

RQ2. What are the associations between mental health literacy and medication-related beliefs and medication adherence, and to what extent do these psychological and cognitive factors explain the variability in medication adherence?

### 3.2. Associations Between Psychological–Cognitive Factors and Medication Adherence

Medication adherence was positively associated with mental health literacy (ρ = 0.361, 95% CI [0.239, 0.476], *p* < 0.001) and necessity beliefs (ρ = 0.353, 95% CI [0.234, 0.458], *p* < 0.001) and negatively associated with medication concerns (ρ = −0.474, 95% CI [−0.571, −0.366], *p* < 0.001), perceived medication harm (ρ = −0.528, 95% CI [−0.616, −0.426], *p* < 0.001), and perceived medication overuse (ρ = −0.284, 95% CI [−0.397, −0.161], *p* < 0.001). Perceived harm to medication had the strongest bivariate association with adherence (Table 2).

In the hierarchical regression, mental health literacy alone explained 9.3% of the variance in medication adherence (R^2^ = 0.093, adjusted R^2^ = 0.089, *p* < 0.001). Adding the four medication belief dimensions increased the explained variance to 40.0% (R^2^ = 0.400, adjusted R^2^ = 0.386), representing a significant incremental contribution of 30.7% (ΔR^2^ = 0.307, ΔF (4, 219) = 28.00, *p* < 0.001). In the fully adjusted model, greater mental health literacy (β = 0.205, *p* < 0.001) and stronger necessity beliefs (β = 0.228, *p* < 0.001) were independently associated with greater adherence, whereas medication concerns (β = −0.354, *p* < 0.001) and perceived medication harm (β = −0.171, *p* = 0.011) were associated with lower adherence. Perceived medication overuse was not independently associated with adherence (β = −0.029, *p* = 0.579). Medication concerns showed the largest standardized independent association (Table 3).

**Figure 2:**
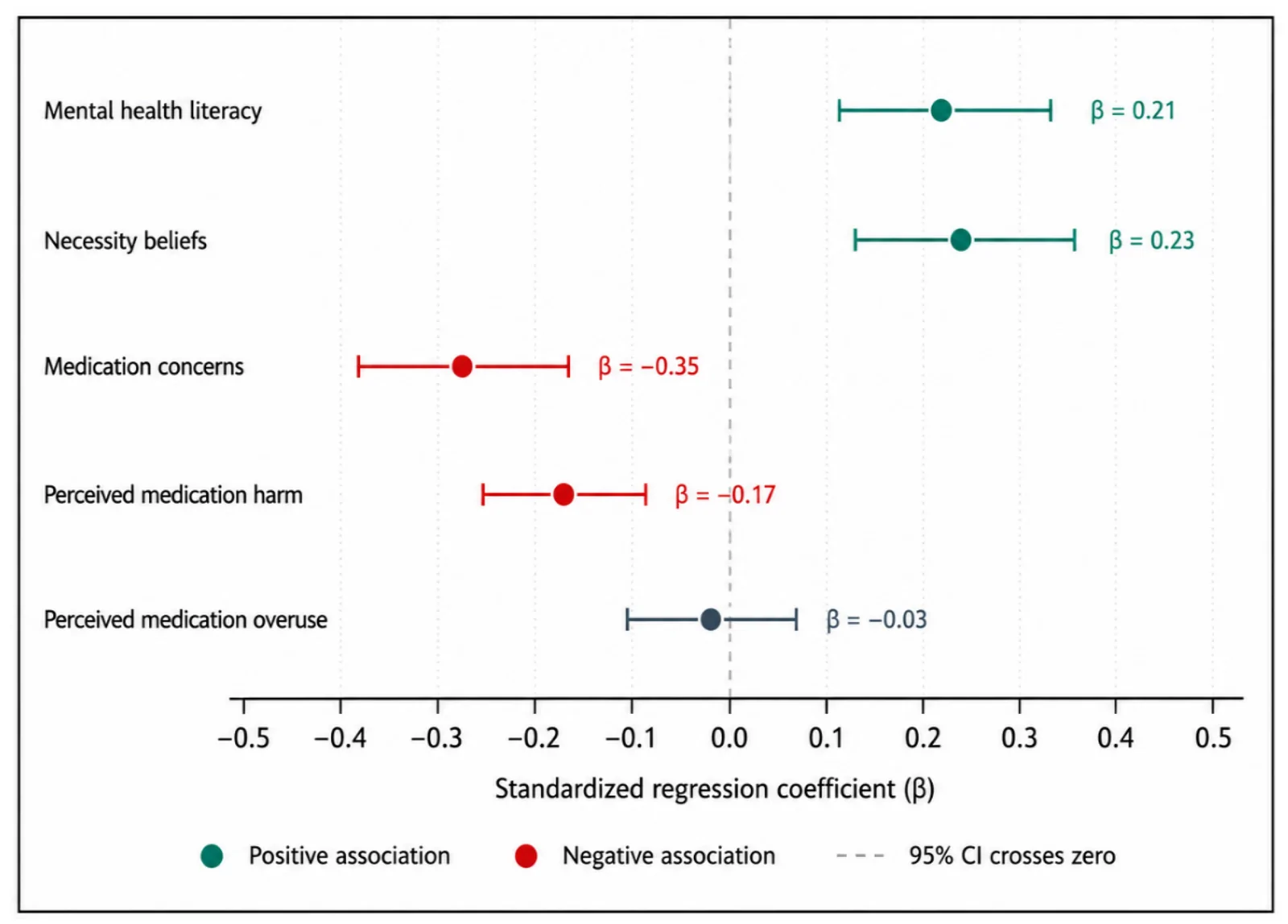
Standardized regression coefficients (β) and 95% confidence intervals for psychological–cognitive factors associated with medication adherence. Confidence intervals were estimated using 5000 bootstrap resamples (*n* = 225).

Regression diagnostics indicated no problematic multicollinearity (maximum VIF = 1.80) or substantial violations of heteroscedasticity or residual normality (Breusch–Pagan *p* = 0.112; Jarque–Bera *p* = 0.136). Nevertheless, HC3 robust standard errors were retained for conservative inference. The standardized coefficients and their 95% confidence intervals are shown in Figure 2.

RQ3. To what extent can medication adherence be predicted using psychological–cognitive, sociodemographic, and clinical characteristics, and does an integrated machine-learning model improve out-of-sample predictive performance?

#### Machine-Learning Prediction of Medication Adherence

Machine learning models were evaluated using three feature sets: sociodemographic/clinical, psychological–cognitive, and integrated predictors. The Elastic Net, Random Forest, and Gradient Boosting regression models were compared using repeated five-fold cross-validation. To ensure direct comparability across feature sets, analyses were conducted on participants with complete data for all predictors (*n* = 208). The predictive performance was evaluated using the cross-validated R^2^, mean absolute error (MAE), and root mean squared error (RMSE).

As shown in Table 4, models based solely on sociodemographic and clinical characteristics showed limited cross-validated predictive performance (R^2^ = −0.002 to 0.086), whereas psychological–cognitive models performed substantially better (R^2^ = 0.339–0.375). The integrated Random Forest model achieved the best overall performance (R^2^ = 0.402, MAE = 0.958, RMSE = 1.299), followed by the integrated Gradient Boosting model (R^2^ = 0.361, MAE = 1.028, RMSE = 1.343). These estimates reflect internal cross-validated performance and should not be interpreted as evidence of prospective or external clinical validity.

**Figure 3:**
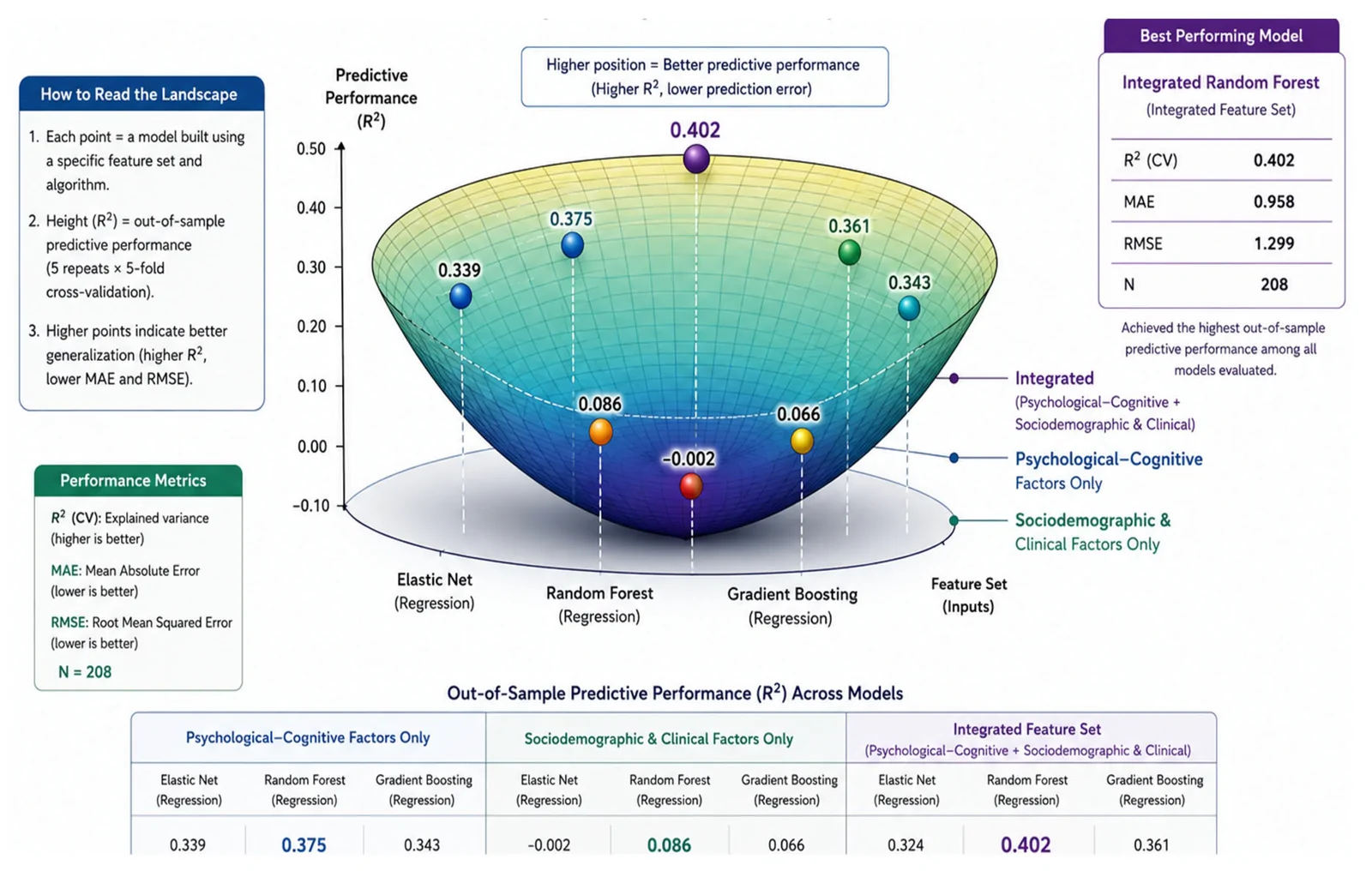
Internal cross-validated predictive performance across machine learning algorithms and feature sets using repeated five-fold cross-validation (five repeats). The integrated Random Forest model achieved the highest cross-validated predictive performance (R^2^ = 0.402, MAE = 0.958, RMSE = 1.299; N = 208).

The difference between the psychological and cognitive Random Forest model (R^2^ = 0.375) and the integrated Random Forest model (R^2^ = 0.402) was modest (ΔR^2^ = 0.027), indicating that most of the cross-validated predictive information was attributable to psychological–cognitive factors, with sociodemographic and clinical characteristics providing limited additional predictive value. The comparative cross-validated predictive performance of the evaluated machine-learning models and feature sets is presented in Figure 3.

RQ4. Which psychological, cognitive, sociodemographic, and clinical factors contribute most strongly to machine learning predictions of medication adherence, and how do they influence model predictions?

The SHAP-based importance of the predictors in the best-performing integrated Random Forest model is presented in Table 5.

SHAP analysis of the best-performing integrated Random Forest model identified substantial differences in the relative contribution of individual predictors to out-of-sample medication adherence predictions. Perceived medication harm (Table 6) was the most influential predictor (mean |SHAP| = 0.537, 32.6%), followed by medication concerns (0.350, 21.2%), mental health literacy (0.195, 11.8%), and necessity beliefs (0.138, 8.4%). Together, these four psychological and cognitive factors accounted for approximately 74% of the aggregated SHAP importance.

**Figure 4:**
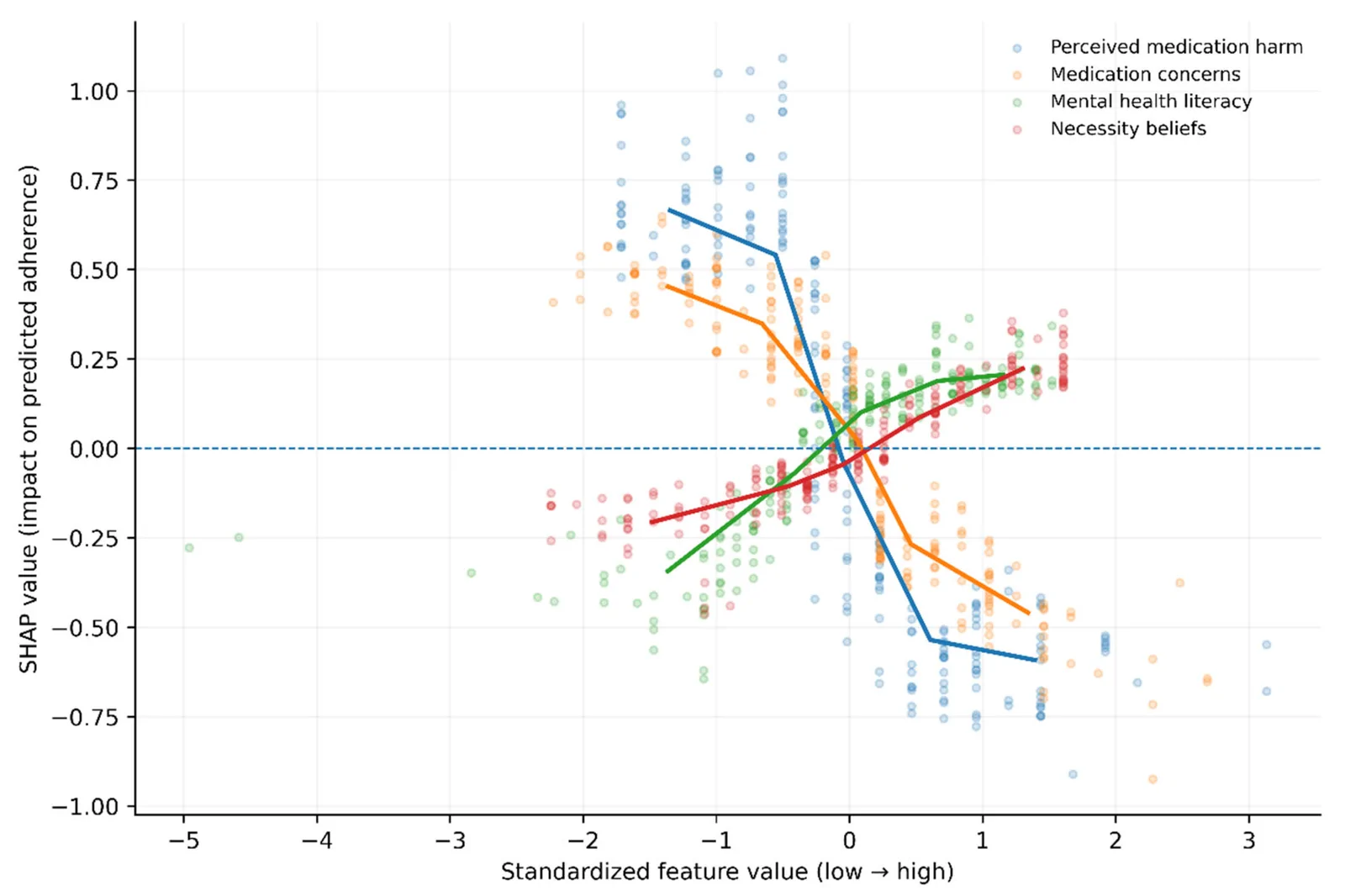
SHAP summary plot for the integrated Random Forest model. Positive SHAP values indicate contributions toward higher predicted medication adherence, and negative values toward lower predicted adherence; point colors represent feature values from low to high. SHAP = SHapley Additive exPlanations; *n* = 208.

Among the sociodemographic and clinical predictors, age (mean |SHAP| = 0.073; 4.4%) and educational level (0.068; 4.1%) showed the largest contributions, although both were substantially less influential than the leading psychological–cognitive predictors. The SHAP patterns indicated that mental health literacy, necessity beliefs, medication concerns, and perceived medication harm were the principal contributors to the individual model predictions. These SHAP values describe the feature contributions within the fitted model and should not be interpreted as causal effects. Overall, the SHAP patterns were broadly consistent with conventional association analyses, while the directional SHAP contributions of the leading predictors are illustrated in Figure 4.

RQ5. Are the associations between mental health literacy and medication adherence statistically accounted for, in part, by medication-related beliefs?

**Parallel:** Indirect-Effects Analysis.

Mental health literacy was positively associated with medication adherence, both directly and indirectly, through the selected medication-belief dimensions. In the parallel indirect-effects analysis, statistically supported specific indirect associations were observed through necessity beliefs (indirect effect = 0.0075, 95% bootstrap CI [0.0019, 0.0153]) and perceived medication harm (indirect effect = 0.0081, 95% bootstrap CI [0.0013, 0.0181]). In contrast, indirect associations through medication concerns (indirect effect = 0.0056, 95% bootstrap CI [−0.0037, 0.0176]) and perceived medication overuse (indirect effect = −0.0002, 95% bootstrap CI [−0.0028, 0.0018]) were not statistically supported. The total indirect association was statistically supported (indirect effect = 0.0210, 95% bootstrap CI [0.0051–0.0398]). The direct association between mental health literacy and medication adherence remained positive after the simultaneous inclusion of the four medication-belief dimensions (B = 0.0431, 95% bootstrap CI [0.0219, 0.0694]), indicating that the association was only partially statistically accounted for by medication-related beliefs. Given the cross-sectional design, these findings were interpreted as statistically indirect associations rather than evidence of temporal or causal mediation (Table 7).

**Figure 5:**
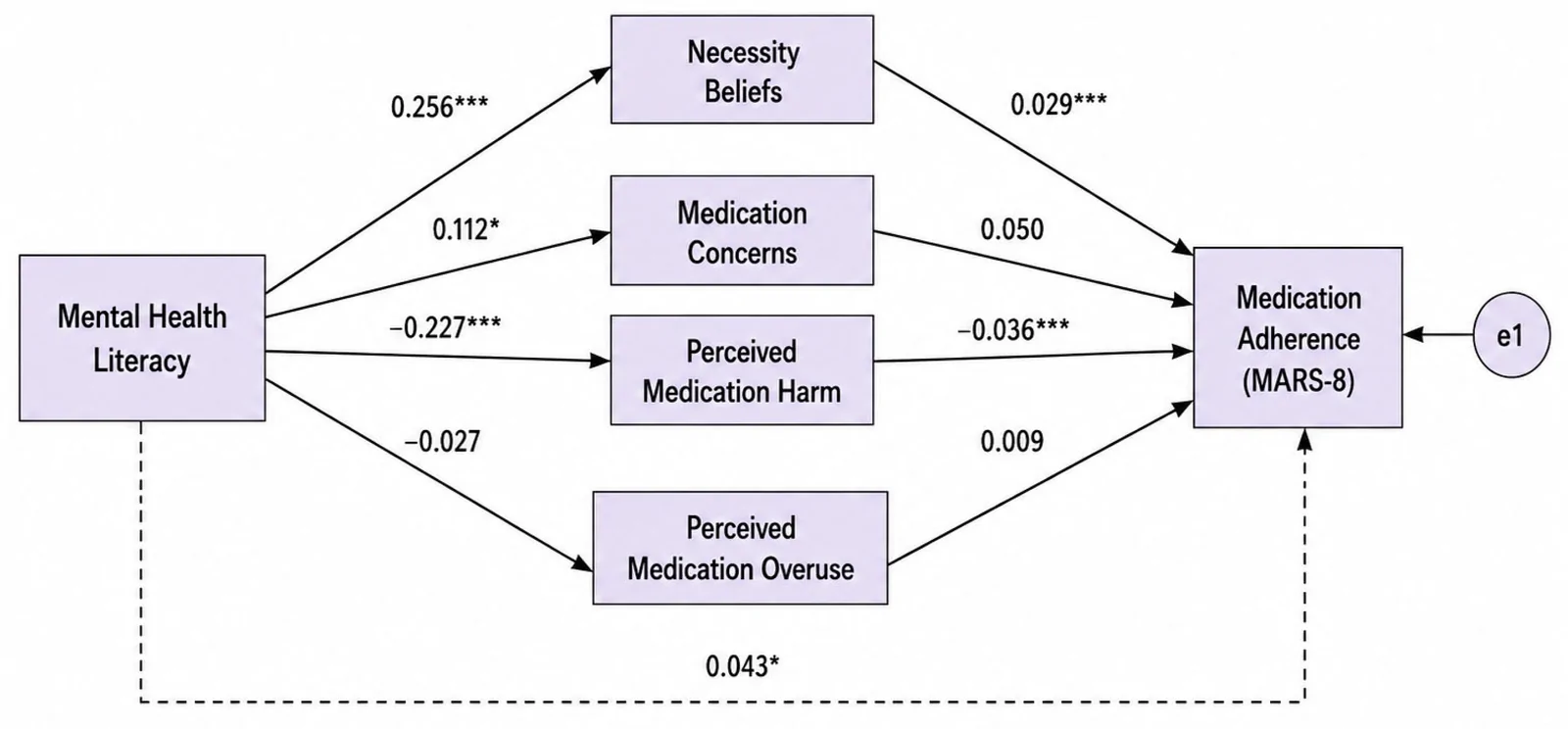
Parallel indirect effects model of the association between mental health literacy and medication adherence through medication-related belief dimensions. Unstandardized path coefficients are shown. The dashed path represents the direct association between mental health literacy and medication adherence after the simultaneous inclusion of the four intervening variables. The indirect effects and their 95% bootstrap confidence intervals are reported in Table 8. *n* = 225. * *p* < 0.05; *** *p* < 0.001.

Figure 5 provides a graphical representation of the parallel indirect effects model. Mental health literacy was specified as the predictor, the four medication belief dimensions as parallel intervening variables, and medication adherence as the outcome. Standardized path estimates are displayed together with the direct association between mental health literacy and medication adherence after the simultaneous inclusion of the four medication-belief dimensions.

**Table 8:** Bootstrap Estimates of Indirect Associations Between Mental Health Literacy and Medication Adherence Through Medication-Related Belief Dimensions.

Pathway	Indirect Effect (B)	95% Bootstrap CI	Interpretation
MHL → Necessity → Adherence	0.0075	[0.0019, 0.0153]	Supported
MHL → Concerns → Adherence	0.0056	[−0.0037, 0.0176]	Not supported
MHL → Harm → Adherence	0.0081	[0.0013, 0.0181]	Supported
MHL → Overuse → Adherence	−0.0002	[−0.0028, 0.0018]	Not supported
Total indirect association	0.0210	[0.0051, 0.0398]	Supported

Note. MHL = mental health literacy; CI = confidence interval. Confidence intervals were estimated using 5000 bootstrap resamples. An indirect association was considered statistically supported when its 95% bootstrap CI did not include zero. As the data were cross-sectional, indirect associations should not be interpreted as evidence of temporal or causal mediation.

## 4. Discussion

The present study identified consistent associations between psychological–cognitive factors and the primary MARS-derived 8-item adherence composite. Greater mental health literacy and stronger necessity beliefs were associated with higher adherence composite scores, whereas medication concerns and perceived medication harm were associated with lower scores. Psychological–cognitive variables accounted for substantially more explanatory and predictive information than did sociodemographic and clinical characteristics. However, these findings should be interpreted in light of the measurement properties of the primary adherence outcome and sensitivity analyses conducted to distinguish behavioral adherence from attitudinal and experiential medication content.

Importantly, sensitivity analyses materially qualified the magnitude of the primary associations. When adherence was restricted to the available behavioral items assessing medication forgetting and carelessness, the directions of most associations were retained but were substantially attenuated. The psychological–cognitive predictors explained 8.0% of the variance in the behavioral adherence score (R^2^ = 0.080) compared with 40.0% of the variance in the primary eight-item composite (R^2^ = 0.400). This attenuation suggests that the comparatively strong associations observed for the primary composite may partly reflect shared attitudinal content between the adherence outcome and medication-belief predictors [6,14]. Accordingly, the MARS-derived 8-item score should be interpreted as a broader adherence-related composite incorporating medication-taking behavior together with medication-related attitudes and experiences, rather than as a purely behavioral measure of medication adherence [23].

### 4.1. Mental Health Literacy and Medication Adherence

Mental health literacy was positively associated with the primary MARS-derived 8-item adherence composite, although its contribution was modest relative to medication-related beliefs. This finding is consistent with the broader health literacy literature suggesting that a greater understanding of mental health conditions and treatment may facilitate informed engagement with pharmacotherapy [1,25,26]. However, the behavioral sensitivity analysis qualified this association: mental health literacy showed only a small positive correlation with the two-item behavioral adherence score (ρ = 0.132, *p* = 0.048) and was not independently associated with behavioral adherence after simultaneous adjustment for medication belief dimensions (β = 0.104, *p* = 0.136). Accordingly, the present findings support an association between mental health literacy and the broader adherence-related composite, but provide more limited evidence for an independent relationship with medication-taking behavior itself.

This distinction is clinically relevant because mental health literacy and medication beliefs represent related but conceptually distinct domains. Patients may understand their psychiatric condition and treatment while simultaneously holding concerns about medication necessity, harm, or overuse. Mental health literacy should therefore be interpreted as a potentially enabling contextual factor rather than a sufficient determinant of medication-taking behavior [24]. Given the cross-sectional design, causal or directional interpretations cannot be established, and prospective studies using validated behavioral adherence measures are needed to clarify whether improvements in mental health literacy translate into sustained medication-taking behavior.

### 4.2. Necessity Beliefs and Medication Adherence

Necessity beliefs were positively associated with the primary MARS-derived 8-item adherence composite, consistent with the Necessity–Concerns Framework, which conceptualizes medication-taking decisions in relation to patients’ perceived personal need for treatment and concerns about its potential adverse consequences [14]. Previous evidence has similarly linked stronger necessity beliefs to better medication adherence across long-term conditions [16,19]. However, the behavioral sensitivity analysis substantially qualified this finding. Necessity beliefs showed only a small positive association with the two-item behavioral adherence score (ρ = 0.131, *p* = 0.049) and were not independently associated with behavioral adherence after simultaneous adjustment for other psychological–cognitive predictors (β = 0.116, *p* = 0.118). Thus, the present findings support a relationship between necessity beliefs and the broader adherence-related composite but provide more limited evidence that necessity beliefs independently predict medication-taking behavior itself. Clinically, perceived necessity remains relevant to understanding patients’ evaluations of psychotropic treatment; however, its behavioral implications should be interpreted cautiously and examined prospectively using validated behavioral adherence measures.

### 4.3. Negative Medication Beliefs and Adherence

Negative medication beliefs showed some of the strongest associations with the primary MARS-derived 8-item adherence composite. Perceived medication harm had the strongest bivariate association with the primary composite, whereas medication concerns showed a prominent independent association in the multivariable analysis. These findings are broadly consistent with previous evidence linking concerns about medication, perceived adverse consequences, and negative treatment evaluations to poorer medication adherence [14,16,19]. However, behavioral sensitivity analysis substantially qualified these relationships. Medication concerns remained negatively associated with the two-item behavioral adherence score at the bivariate level (ρ = −0.184, *p* = 0.006), but the adjusted association did not reach conventional statistical significance (β = −0.147, *p* = 0.065). Perceived medication harm was not significantly associated with behavioral adherence in either the bivariate analysis (ρ = −0.128, *p* = 0.055) or the adjusted model (β = 0.103, *p* = 0.287).

Notably, perceived medication overuse exhibited a different pattern. Although it was not the strongest correlate of the primary eight-item composite, it demonstrated the largest negative bivariate association with the behavioral adherence score among the medication belief dimensions (ρ = −0.233, *p* < 0.001) and was the only medication belief predictor that remained independently associated with poorer behavioral adherence in the sensitivity model (β = −0.185, 95% CI [−0.340, −0.030], *p* = 0.019). Taken together, these findings indicate that the relative importance of specific medication beliefs depends on how adherence is operationalized. Therefore, the stronger associations observed with the broader MARS-derived composite should not be interpreted as equivalent evidence of effects on medication-taking behavior, particularly given the attitudinal and experiential content of the primary outcome [20,23].

### 4.4. Interpretation of Machine-Learning Predictive Performance

The machine learning findings provide an important extension beyond conventional association analysis. Models relying solely on sociodemographic and clinical variables showed limited out-of-sample predictive performance, whereas models incorporating psychological–cognitive variables performed substantially better. The integrated Random Forest model achieved the highest cross-validated predictive performance (R^2^ = 0.402), although its improvement over the psychological–cognitive Random Forest model (R^2^ = 0.375) was modest. Thus, most of the predictive signals appeared to originate from psychological–cognitive information, with sociodemographic and clinical variables providing comparatively limited incremental value [25].

This result is important because medication adherence prediction has increasingly incorporated machine-learning methods; however, previous research has been highly heterogeneous in predictors, outcomes, and validation strategies. Bohlmann et al. [27], in a scoping review of 43 studies, highlighted both the potential of machine learning for adherence-related applications and the methodological diversity of the field. The present findings suggest that improving prediction may depend not merely on increasing the number of available clinical variables but also on incorporating theoretically meaningful and potentially modifiable patient-level constructs [28]. Nevertheless, the observed predictive performance should not be interpreted as the establishment of clinical readiness. Although repeated cross-validation provides stronger evidence than in-sample performance, models require external validation using independent psychiatric samples before clinical application. The current findings therefore demonstrate a predictive signal, rather than a validated clinical prediction tool.

### 4.5. Predictive Modeling and Explainable Machine Learning

SHAP analysis strengthens the predictive findings by identifying the variables that contributed most strongly to model predictions. Perceived harm, medication concerns, mental health literacy, and necessity beliefs jointly accounted for approximately 74% of the aggregated SHAP importance. Their directional contribution was also consistent with conventional analyses: greater literacy and necessity tended to shift predictions toward higher adherence, whereas concerns and harm shifted predictions toward lower adherence [29].

Complementary analyses provided different perspectives on the same underlying associations. Regression quantified adjusted associations, machine learning evaluated out-of-sample predictions, and SHAP characterized the contribution of individual predictors to the model predictions. The secondary path representation descriptively reproduced the multivariable association structure but was not treated as independent confirmatory evidence. SHAP values explain model predictions and should not be interpreted as causal effects [20,29,30].

### 4.6. Secondary Path Representation of Medication Adherence

The secondary path representation reproduced the direction and relative magnitude of the associations observed in the multivariable regression. Because this representation was just identified and based on the same variables and outcomes, it should not be interpreted as independent confirmatory or convergent evidence. Its role was limited to descriptively illustrating the simultaneous psychological–cognitive association structure.

This explanatory capacity is meaningful given the recognized complexity of adherence behavior [31]. A systematic review and meta-analysis of psychotropic medication non-adherence in major psychiatric disorders demonstrated that non-adherence is associated with multiple patient, illness, treatment, and contextual factors rather than a single determinant. Likewise, a systematic review by Marrero and Fumero [6] emphasized the contribution of psychological variables alongside sociodemographic and clinical influences. The present model, therefore, should not be interpreted as a complete account of adherence, but as evidence that psychological–cognitive representations constitute a substantial component within a broader multifactorial process [31,32].

The unexplained variance may reflect factors not fully represented in the present framework, including symptom severity, medication side effects, regimen complexity, cognitive functioning, therapeutic alliance, stigma, accessibility of care, family involvement, socioeconomic constraints, and healthcare system characteristics. Indeed, family support has also been identified as relevant to psychopharmacological adherence in psychiatric research [33]. More importantly, the observed multivariable associations clarify how the variables can be collectively understood. Mental health literacy may provide an informational foundation for understanding illness and treatment, whereas necessity beliefs, concerns, and harm perceptions represent more proximal cognitive appraisals through which patients evaluate whether medication is personally worthwhile. This distinction may explain why simply increasing knowledge is unlikely to resolve non-adherence when strong concerns or negative medication beliefs remain unaddressed [34,35].

## 5. Clinical and Practical Implications

These results support the potential value of a more personalized approach to adherence assessment in psychiatry. Clinicians may obtain more actionable information by assessing not only whether patients take their medication but also whether they understand their treatment, perceive it as personally necessary, and have concerns about harm or adverse consequences. A systematic review similarly indicates that psychological factors, health beliefs, and perceived barriers are important components of psychopharmacological adherence [3,6,35]. Accordingly, psychoeducation should extend beyond factual information. Interventions may be more useful when they combine mental health literacy with individualized discussions of necessity beliefs, medication concerns, perceived harm, and patients’ treatment experiences [36]. The predictive findings further suggest that these potentially modifiable factors may eventually contribute to adherence-risk stratification; however, any such application would require prospective and external validation before clinical implementation.

## 6. Strengths and Limitations

A principal strength of this study is the integration of theoretically grounded psychological constructs with conventional regression, internally cross-validated machine learning prediction, and SHAP-based model interpretation. These complementary analyses addressed distinct analytical purposes rather than providing independent convergent evidence. Repeated cross-validation reduced reliance on apparent in-sample predictive performance, while sensitivity analyses directly examined the robustness of the findings to the alternative operationalization of medication adherence.

This study has several limitations that warrant consideration. The cross-sectional design precludes causal inference, and self-reported adherence may be affected by recall or social desirability bias. The adapted eight-item MARS-derived measure also precluded the application of an unvalidated categorical adherence cutoff. The relatively low internal consistency of the MARS-derived eight-item adherence measure (KR-20/Cronbach’s α = 0.490) is an important limitation. This measurement error may have attenuated the observed associations and may also have affected predictive performance; accordingly, the magnitude of the explanatory and predictive estimates should be interpreted cautiously.

The combination of relatively low internal consistency (KR-20/Cronbach’s α = 0.490) and an R^2^ of 0.400 for the primary eight-item composite should not be interpreted as indicating that the predictors explain approximately 82% of the independently estimated “true-score” variance. This ratio assumes a classical unidimensional measurement model that is not warranted for this heterogeneous MARS-derived composite. Rather, the comparatively high R^2^ may partly reflect the shared attitudinal and experiential content between the outcome and medication belief predictors. Consistent with this interpretation, when the outcome was restricted to the two available behavioral adherence items, the explained variance decreased markedly to R^2^ = 0.080. Accordingly, the primary R^2^ should be interpreted as the variance explained in the observed broader adherence-related composite, not as an estimate of variance explained in a latent error-free medication-adherence construct. Recruitment from a single psychiatric hospital may also limit the generalizability of the findings. Machine learning analyses were restricted to 208 complete cases and require replication in larger independent samples. Finally, SHAP explains model behavior rather than causal mechanisms, and the predictive models were internally evaluated but not externally validated. These limitations support cautious interpretation of the models as explanatory and predictive evidence rather than clinically deployable tools.

## 7. Future Research Directions

Future research should prioritize longitudinal, multicenter, and externally validated designs. Prospective studies are needed to determine whether changes in mental health literacy, necessity beliefs, concerns, and perceived harm precede subsequent changes in adherence. Larger samples would also permit external validation, calibration assessment, diagnostic subgroup analyses, and evaluation of additional predictors, such as adverse effects, therapeutic alliance, family influences, and treatment history. Most importantly, intervention studies should test whether modifying the psychological–cognitive factors identified here leads to measurable improvements in medication adherence.

## 8. Conclusions

Medication adherence among psychiatric patients appears to be meaningfully associated with an interconnected set of psychological–cognitive factors. Greater mental health literacy and stronger beliefs about the necessity of prescribed medication were associated with better adherence, whereas medication concerns and perceptions of medication-related harm were associated with poorer adherence. Medication concerns emerged as a particularly influential factor across the analytical framework, while perceived medication overuse contributed little after the remaining factors were considered simultaneously.

Complementary conventional and predictive analyses indicated that psychological and cognitive factors provided substantial information about medication adherence beyond sociodemographic and clinical characteristics alone. The secondary path representation descriptively illustrated these multivariable associations and was not interpreted as independent confirmatory evidence. These findings support patient-centered adherence strategies that combine mental health literacy enhancement with systematic identification and discussion of medication-related beliefs and concerns. Nevertheless, predictive findings require prospective replication and external validation before clinical implementation, while path representation should be regarded as descriptive rather than confirmatory.

## Figures and Tables

**Table 1:** Sociodemographic and Clinical Characteristics of the Study Participants (N = 225).

Characteristic	Category	*n*	%
Age (years)	25–30	56	24.9
	31–36	63	28.0
	37–42	59	26.2
	43–47	47	20.9
Sex	Male	133	59.1
	Female	92	40.9
Marital status	Single	98	43.6
	Married	96	42.7
	Divorced	27	12.0
	Widowed	4	1.8
Educational level	Primary	13	5.8
	Intermediate	11	4.9
	Secondary	97	43.1
	University	96	42.7
	Postgraduate	8	3.6
Employment status	Employed	92	40.9
	Unemployed	113	50.2
	Retired	14	6.2
	Self-employed	6	2.7
Economic level	Low	82	36.4
	Moderate	132	58.7
	High	11	4.9
Residence	Urban	158	70.2
	Rural/Village	64	28.4
	Bedouin area	3	1.3
Psychiatric diagnosis	Depressive and mood disorders	106	47.1
	Anxiety disorders	52	23.1
	Schizophrenia and other psychotic disorders	22	9.8
	Personality disorders	19	8.4
	Substance use/addiction disorders	16	7.1
	Organic psychiatric disorders	10	4.4
Duration of psychiatric disorder	<1 year	37	16.4
	1–5 years	110	48.9
	>5 years	78	34.7
Number of psychotropic medications	One medication	81	36.0
	Two medications	89	39.6
	≥3 medications	55	24.4
Family support	Present	149	66.2
	Absent	76	33.8
Treatment follow-up	Regular	196	87.1
	Irregular	29	12.9

**Table 3:** Item-Level Psychometric Properties of the MARS-Derived 8-Item Adherence Measure.

Item	Adherence-Consistent Response (%)	Corrected Item–Total Correlation	α If Item Deleted
Forgetting to take medication	39.1	0.105	0.504
Carelessness about taking medication	71.6	0.282	0.432
Taking medication only when feeling ill	79.6	0.272	0.439
Feeling that medication unnaturally controls mind and body	56.9	0.165	0.481
Thoughts being clearer when taking medication	71.6	0.261	0.440
Medication preventing recurrence of illness	82.7	0.175	0.473
Feeling “like a zombie” when taking medication	72.0	0.222	0.456
Medication causing tiredness or sluggishness	52.0	0.309	0.418

Note. *n* = 225. The items were coded in an adherence-consistent direction. Corrected item–total correlations were calculated between each item and the sum of the remaining items. The internal consistency of the complete eight-item measure was KR-20/Cronbach’s α = 0.490.

**Table 4:** Associations Between Medication Adherence and Psychological–Cognitive Factors.

Variable	Spearman’s ρ	95% CI	*p*
Mental health literacy	0.361	[0.239, 0.476]	<0.001
Necessity beliefs	0.353	[0.234, 0.458]	<0.001
Medication concerns	−0.474	[−0.571, −0.366]	<0.001
Perceived medication harm	−0.528	[−0.616, −0.426]	<0.001
Perceived medication overuse	−0.284	[−0.397, −0.161]	<0.001

Note. *n* = 225. Higher medication adherence scores indicated greater adherence. Confidence intervals were estimated using 5000 bootstrap resamples.

**Table 5:** SHAP-Based Importance of the Leading Predictors in the Integrated Random Forest Model.

Rank	Feature	Mean |SHAP|	Relative Importance
1	Perceived medication harm	0.537	32.6%
2	Medication concerns	0.350	21.2%
3	Mental health literacy	0.195	11.8%
4	Necessity beliefs	0.138	8.4%
5	Age	0.073	4.4%
6	Educational level	0.068	4.1%
7	Sex	0.046	2.8%
8	Psychiatric diagnosis	0.045	2.7%
9	Number of psychotropic medications	0.034	2.0%
10	Perceived medication overuse	0.032	1.9%

**Table 6:** Hierarchical Multiple Regression Analysis of Psychological–Cognitive Factors Associated with Medication Adherence.

Model/Predictor	B	Robust SE	β	95% CI for B	*p*
Model 1					
Mental health literacy	0.064	0.016	0.305	[0.032, 0.096]	<0.001
Model 2					
Mental health literacy	0.043	0.012	0.205	[0.019, 0.067]	<0.001
Necessity beliefs	0.074	0.018	0.228	[0.039, 0.110]	<0.001
Medication concerns	−0.122	0.020	−0.354	[−0.160, −0.084]	<0.001
Perceived medication harm	−0.071	0.028	−0.171	[−0.125, −0.016]	0.011
Perceived medication overuse	−0.019	0.034	−0.029	[−0.085, 0.047]	0.579

Note. *n* = 225. B = unstandardized regression coefficient; robust SE = HC3 heteroskedasticity-consistent standard error; β = standardized regression coefficient; CI = confidence interval. Model 1: R^2^ = 0.093, adjusted R^2^ = 0.089. Model 2: R^2^ = 0.400, adjusted R^2^ = 0.386; ΔR^2^ = 0.307, ΔF(4, 219) = 28.00, *p* < 0.001.

**Table 7:** Cross-Validated Performance of Machine-Learning Models for Predicting Medication Adherence.

Feature Set	Algorithm	Mean CV *R*^2^	MAE	RMSE
Sociodemographic/clinical	Elastic Net	−0.002	1.374	1.693
Sociodemographic/clinical	Random Forest	0.086	1.253	1.617
Sociodemographic/clinical	Gradient Boosting	0.066	1.301	1.632
Psychological–cognitive	Elastic Net	0.339	1.095	1.368
Psychological–cognitive	Random Forest	0.375	1.002	1.329
Psychological–cognitive	Gradient Boosting	0.343	1.047	1.363
Integrated	Elastic Net	0.324	1.099	1.384
Integrated	Random Forest	0.402	0.958	1.299
Integrated	Gradient Boosting	0.361	1.028	1.343

Note. *n* = 208. CV = cross-validation; MAE = mean absolute error; RMSE = root mean squared error. Performance estimates were obtained using repeated five-fold cross-validation (five repeats).

## Data Availability

The data presented in this study are available on request from the corresponding author due to ethical and privacy restrictions related to the protection of confidential information of psychiatric patients.

## Supplementary Materials

The following supporting information can be downloaded at: https://www.mdpi.com/article/10.3390/healthcare14172777/s1, Table S1: Administered Items, Domains, and Scoring of the 16-Item Mental Health Literacy Questionnaire–Short Version for Adults (MHLq-SVa).
