# Supplementary material for: Integrated Psychological–Behavioral Predictive Model Using Explainable Machine Learning
Source: Healthcare (Basel). 2026 Sep 1;14(17):2777. doi: 10.3390/healthcare14172777 (PMC13565559; doi:10.3390/healthcare14172777)
Supplement: Supplementary file 1 [file healthcare-14-02777-s001.zip › healthcare-4530108-supplementary.pdf]

Supplementary Table S1. Administered Items, Domains, and Scoring of the 16-Item Mental Health Literacy Questionnaire–Short Version for Adults (MHLq-SVa)

No.	Data item (as administered)	MHLS-SVa item	Dimension	Scoring direction
1	Physical exercise contributes to good mental health	MHLq1	Self-help strategies	Direct
2	Mental disorders do not affect people's behaviors	MHLq6	Erroneous beliefs/stereotypes	**Reverse**
3	Sleeping well contributes to good mental health	MHLq7	Self-help strategies	Direct
4	Seek help from a psychologist	MHLq8	Help-seeking/first aid	Direct
5	Mental disorders do not affect people's feelings	MHLq13	Erroneous beliefs/stereotypes	**Reverse**
6	Only adults have mental disorders	MHLq15	Erroneous beliefs/stereotypes	**Reverse**
7	Changes in brain function may lead to mental disorders	MHLq16	Knowledge	Direct
8	Encourage someone close to seek professional psychological/psychiatric help	MHLq17	Help-seeking/first aid	Direct
9	A balanced diet contributes to good mental health	MHLq19	Self-help strategies	Direct
10	Loss of interest/pleasure is a symptom of depression	MHLq20	Knowledge	Direct
11	Symptom duration is important for diagnosis	MHLq22	Knowledge	Direct
12	Mental disorders affect people's thoughts	MHLq25	Knowledge	Direct
13	Doing something enjoyable contributes to good mental health	MHLq26	Self-help strategies	Direct
14	A person with schizophrenia may see/hear things others do not	MHLq27	Knowledge	Direct
15	Highly stressful situations may cause mental disorders	MHLq28	Knowledge	Direct
16	Seek help from a psychiatrist	MHLq29	Help-seeking/first aid	Direct

Note. Items were scored from 1 (strongly disagree) to 5 (strongly agree). Items 2, 5, and 6 were reverse-scored. Scores were summed to yield a total ranging from 16 to 80; higher scores indicated greater mental health literacy. N = 225; Cronbach's α = .797. Direct = scored as administered; Reverse = reverse-scored before computing the total score.
